# Supplementary material for: Coherent combining of self-cleaned multimode beams
Source: Sci Rep. 2020 Nov 24;10:20481. doi: 10.1038/s41598-020-77505-0 (PMC7686367; doi:10.1038/s41598-020-77505-0)
Supplement: Supplementary file 1 — Supplementary Information. [file 41598_2020_77505_MOESM1_ESM.docx]

Supplementary information for **“Coherent combining of self-cleaned multimode beams”**

Marc Fabert^1^, Maria Săpânțan^1^, Katarzyna Krupa^2^, Alessandro Tonello^1^, Yann Leventoux^1^, Sébastien Février^1^, Tigran Mansurian^1^, Alioune Niang^3^, Benjamin Wetzel^1^, Guy Millot^4,5^, Stefan Wabnitz^6,7^ and Vincent Couderc^1,*^

^1^ Université de Limoges, XLIM, UMR CNRS 7252, 123 Avenue A. Thomas, 87060 Limoges, France

^2^Institute of Physical Chemistry, Polish Academy of Sciences, Warsaw, Poland

*^3^ Dipartimento di Ingegneria dell’Informazione, Università di Brescia, via Branze 38, 25123, Brescia, Italy*

*^4^ Université de Bourgogne Franche-Comté, ICB, UMR CNRS 6303, 9 Avenue A. Savary, 21078 Dijon, France*

*^5^ Institut Universitaire de France (IUF), 1 rue Descartes, 75005 Paris, France*

*^6^ DIET, Sapienza University of Rome Via Eudossiana 18, 00184 Rome, Italy*

*^7^ Novosibirsk State University, Pirogova 1, Novosibirsk 630090, Russia*

e-mail* [Vincent.couderc@xlim.fr](mailto:Vincent.couderc@xlim.fr)

**Spatiotemporal fringes analysis obtained between two self-cleaned beams**

In order to study more accurately the dependence of the fringe contrast on the transverse mode content of the beam, we divided the spectrogram in three different spectral sections, and extracted, for each section separately, the corresponding maximum of the interference pattern modulation. The results are illustrated in panel “b” of Fig. SM1; each curve is obtained by averaging the fringe pattern across the corresponding spectral window only. As we can see, both in the top and in the bottom panels of Fig. SM1b the contrast of the interferometric pattern modulation exceeds 66 %. To show the maximum level of available contrast, in the inset of Fig. SM1 we also display a sample transverse trace, taken at a fixed spectral position (as indicated by the dashed line in Fig. SM1a): the corresponding curve exhibits a contrast as high as 80%. Such high level of mutual coherence is due to the fact that, in this region, the beam is carried mainly by the fundamental mode of the fibre, with only weak contributions from higher-order modes. On the other hand, the central part of the spectrogram mainly consists of higher-order modes, which leads to the observed degradation of the integrated contrast, hence of the beam coherence (see the middle panel of Fig. SM1b). The local maximum of the integrated contrast (15%) is reached there at the center of the fringe pattern: the reason is probably related to the residual contribution of the fundamental mode, which is still present at these wavelengths. The lateral wings of the spatial axis of the spectrogram show a 6% contrast, which is a signature of significant coherence degradation, induced by multimode propagation under the combined action of nonlinearity, diffraction and dispersion.


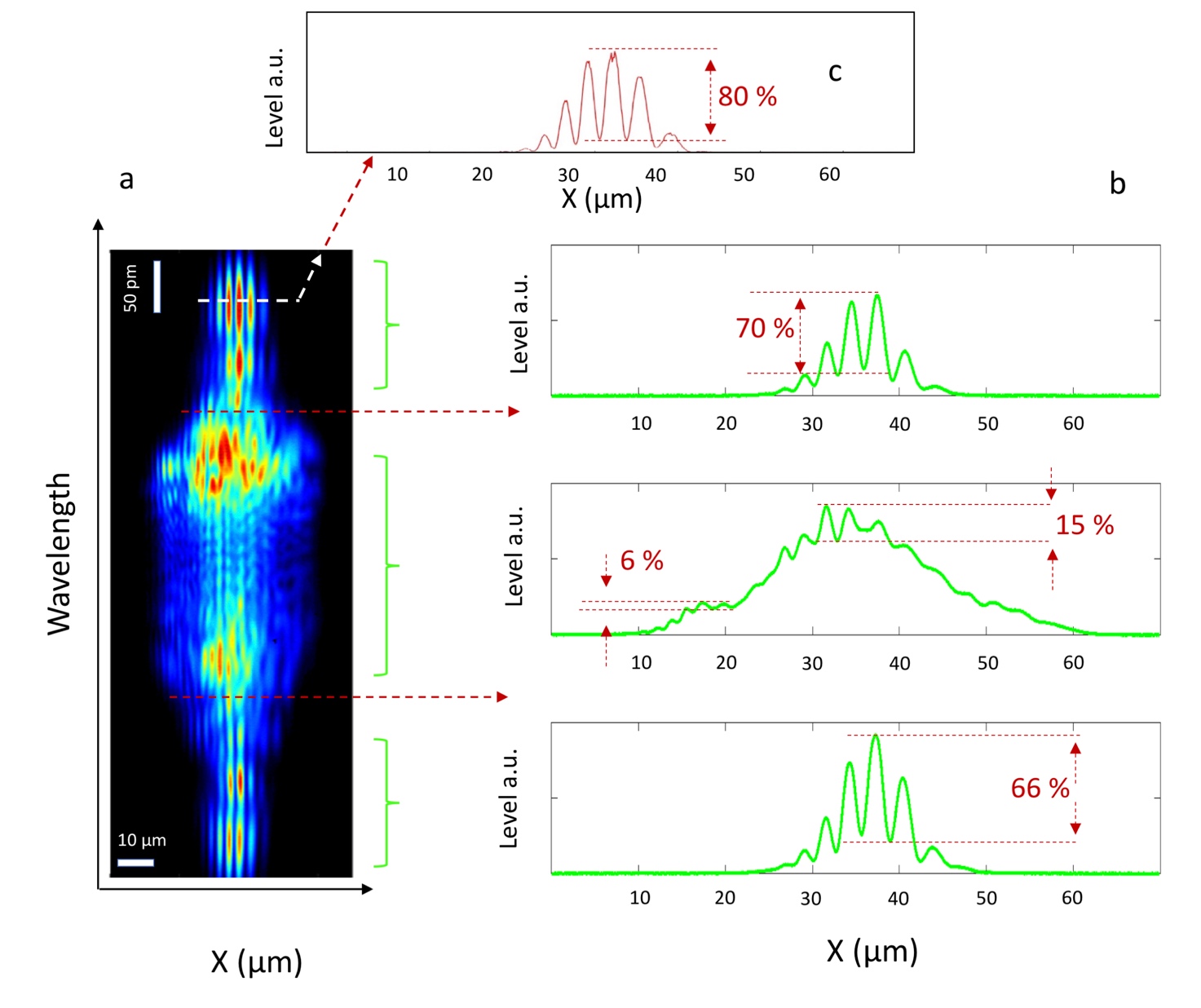


**Figure SM1:** **Wavelength dependence of mutual beam coherence in the beam self-cleaning regime.** a, Spectrogram obtained by dispersing the two interfering self-cleaned beams with an incident angle of ~ 4° (44kW); b, fringe contrast profiles obtained by integration over the relevant wavelength range, and calculated in three different sections of the spectrogram of panel a. c, Sample case of maximum contrast, measured at the spectral position identified by the dashed white line in panel a.

**Fringe contrast evolution upon input peak power**

The pump laser used to observe spatial beam self-cleaning delivers transform-limited pulses with 60 ps pulse duration. We launched laser pulses in two distinct segments of 12-meter-long GRIN MMF. The temporal broadening experienced by the pulses is expected to be a few ps (~1.5 ps). Despite the fact that the beam is guided by many modes (due the highly multimode input excitation condition, and spatial mode coupling upon propagation), at the output of the fibres the two beams preserve a relatively high degree of mutual coherence. As a matter of fact, in a purely linear propagation regime (i.e. at low input peak powers), the interference between the two output speckled beams already exhibits a large contrast (see fig. SM2a); a similar situation is also described in Ref. [1] and references therein. However, fig. SM2b shows that, when we increase the peak power up to the threshold (15 kW) for observing the effect of beam self-cleaning, spectral self- and cross-phase modulation accompanied by parametric FWM processes come into play: all of these processes gradually spoil the mutual coherence, by decreasing the fringe contrast down to 35%. It is important to underline that, in the Kerr beam self-cleaning regime, the fringe contrast can vary significantly, depending on the mode order and wavelength. For instance, the fundamental mode may keep a significant coherent structure with a high contrast in selected frequency windows, whereas the contrast of the remaining speckled background drops down to low values (see results in the main text).

The situation presents some conceptual analogies with the case of phase-locking of high-order harmonic sources described in Ref. [2], and the consequent gradual loss of fringe contrast.


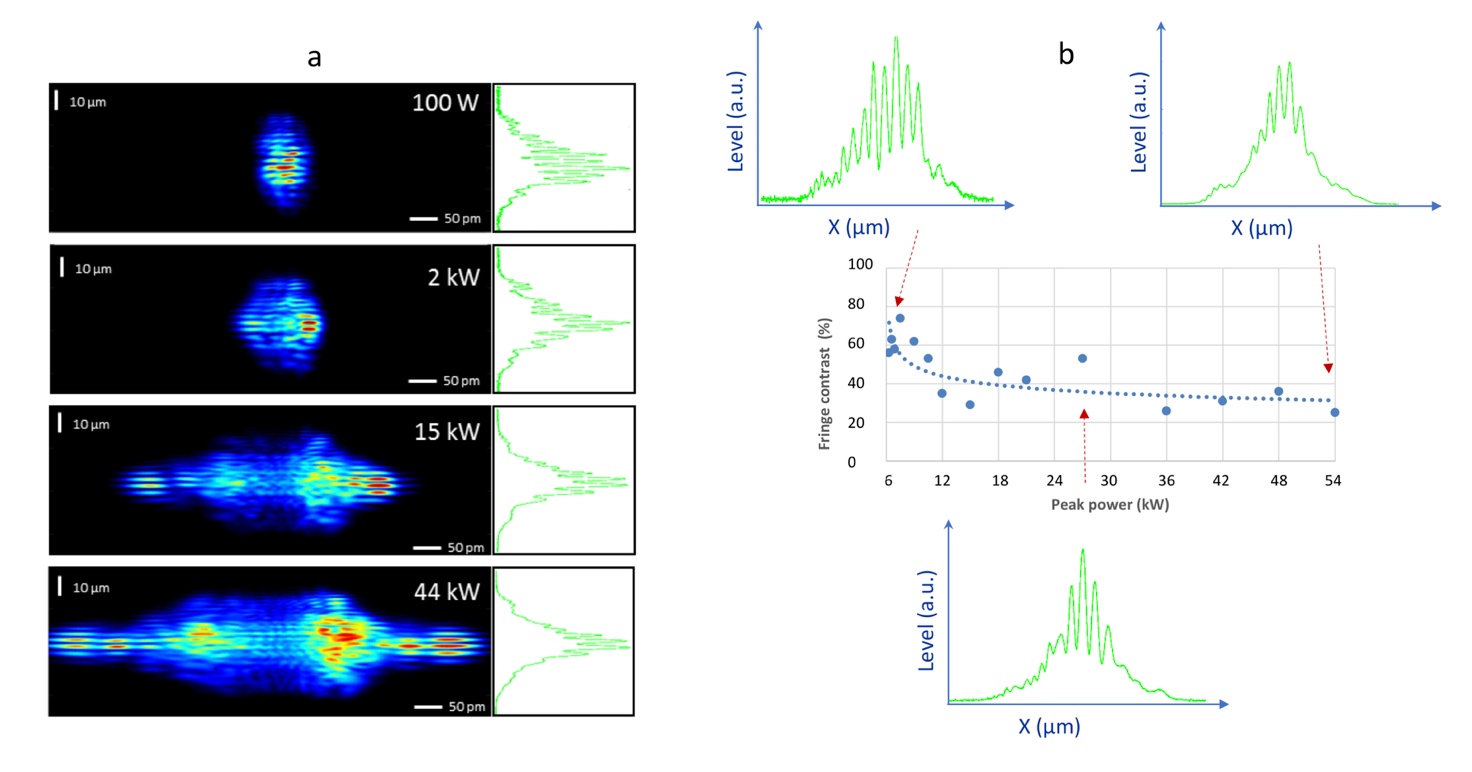


**Figure SM2:** a, Spectrograms, i.e., wavelength resolved interference patterns of two output self-cleaned beams, for various input pump peak powers. Insets: corresponding curve of the transverse spatial profile integrated along the whole available wavelength domain. b, Overall fringe contrast (integrated along the whole spectral domain) versus pump peak power. Insets: examples of selected transverse profiles.

**Additional comments on the self-imaging process**

The self-imaging process observed in GRIN optical fibre has a strong influence on the nonlinear interaction among the modes. It is well understood that when using an input Gaussian beam, it is possible to excite a periodic coherent beating among the propagating modes, with a consequent periodical local increase of the beam intensity. The periodic nature of such coherent beating among the modes suggests also a way to clearly visualize and measure this effect, when the light intensity is particularly high, as it is the case with femtosecond pulses. As a matter of fact, the use of a powerful femtosecond beam can generate local frequency conversions, in the presence of large local beam intensity, with non-guided radiation modes and noncollinear Cerenkov configuration. Then noncollinear converted energy is locally generated in the blue-visible domain, so that the light induced grating can be made visible even at naked eye, although for few tens of centimeters only. The main nonlinear processes at the origin of the observed frequency conversion are: (i) second harmonic generation, with a strong signature at 515 nm (when using a pump 1030 nm); multiphoton absorption by defect states of the Ge-doped fibre, and resulting luminescence [3], producing wideband emission in the blue domain. An example of this light induced grating revealed by nonlinear frequency conversion is given in figure SM3b for the spectrum and in figure SM3c for the longitudinal evolution of the grating. The initial coupling conditions are essential in this problem, as clearly visualized in figure SM3. Normal incidence leads to exciting radially symmetrical modes only: in this case, the local maxima of the light intensity are in the fibre axis. The associated self-imaging period Λ is also reported in the frame SM3a. In the presence of an input tilt, or an off-center shift, the trajectory has a period 2Λ, which is in fact the most general situation for the self-imaging period for a generic input condition, i.e., not limited to radially symmetrical modes. We can also imagine that the presence of a radial symmetry for the light-induced grating will facilitate the coupling among modes with the same symmetry. Whereas the zig-zag trajectory with a tilted input angle will likely facilitate mode coupling with high-order, and antisymmetric modes.

**Self-cleaning of an LP11 mode: a transient effect**

Although spatial self-cleaning has been mainly reported on the fundamental mode of GRIN MMFs, several papers experimentally demonstrated that spatial self-cleaning can be obtained on higher-order modes as well [4-5]. Such particular nonlinear effect has been achieved by properly managing the input coupling conditions, either with an off-center coupling lens [4], or by using a spatial light modulator [5]. In each case, the input power and the fibre length were limited to few tens of kilowatts and few meters, respectively. For the case of an off-center power coupling, mainly odd-parity modes were excited, and an example of the corresponding self-imaging effect [6] is given in fig. SM3c. The same figure also exemplifies the case of a radially symmetrical input beam (see fig. SM3c), as discussed in the previous section. It is reasonable to think that higher-order modes experience a periodic refractive index modulation, which initiates a power exchange between them by means of quasi-phase matched four-wave mixing processes [7]. It is important to note that an irreversible and stable energy distribution between modes was not clearly obtained in these cases. This may be associated with the fact that high-order cleaned mode was not the final state, but it remained subject to further evolution. Hence, the generated high-order mode could be a transient state along the way to an asymptotic spatiotemporal energy repartition between modes (or thermalization). Indeed, the limited fibre length and the particular coupling conditions can drastically modify and drive the energy exchange among the modes, resulting in a particular light path along the fibre. In these conditions, the fibre length that is necessary for observing a full thermalization of the beam can be significantly increased.

In order to prove this point, we carried out both experiments and numerical simulations. In the experiments, we used another laser, namely a sub-nanosecond laser source and a 50/125 GRIN optical fibre. The input beam was tilted by an angle of 2.5°, in order to mainly excite few high-order odd modes (with beam diameter close to 20 µm at full width of half the maximum intensity (FWHMI)). At low input power, we obtained an output speckled beam. Looking at fig. SM4a, we observed that, by increasing the input power, the LP11 mode clearly emerges at the fibre end, and dominates the intensity profile. However, as the powers grows larger, the LP11 mode progressively disappears, in favor of a bell-shaped profile, close to the fundamental mode LP01. One would be then interested to see what is the beam evolution, upon further increases of the input power. However, the exploration of the final stage of the modal evolution is limited by the competing presence of the stimulated Raman scattering process, which grows rapidly after several meters of propagation, and perturbs the evolution of spatial Kerr beam cleaning.

Although it is difficult to carry out a systematic numerical analysis for this effect, a similar evolution can also be seen in a long numerical simulation at a fixed input power, by using the same approach that was developed in ref. [4]. The technique is based on the introduction of a shift of 180° in the input phase plane. In the presence of disorder, the beam is initially fragmented in many speckles. Nevertheless, in subsequent propagation along the fibre, the beam self-organizes into a shape that close to the LP11 mode. However, both the intensity surface at half maximum (integrated over the pulse duration) and the orthogonal projections (calculated in the sample time t=0 at maximum input intensity) show that the LP11 mode subsequently evolves into a different beam shape, with an apparent dominant contribution from the fundamental mode (see fig. SM4b and SM4c).

**
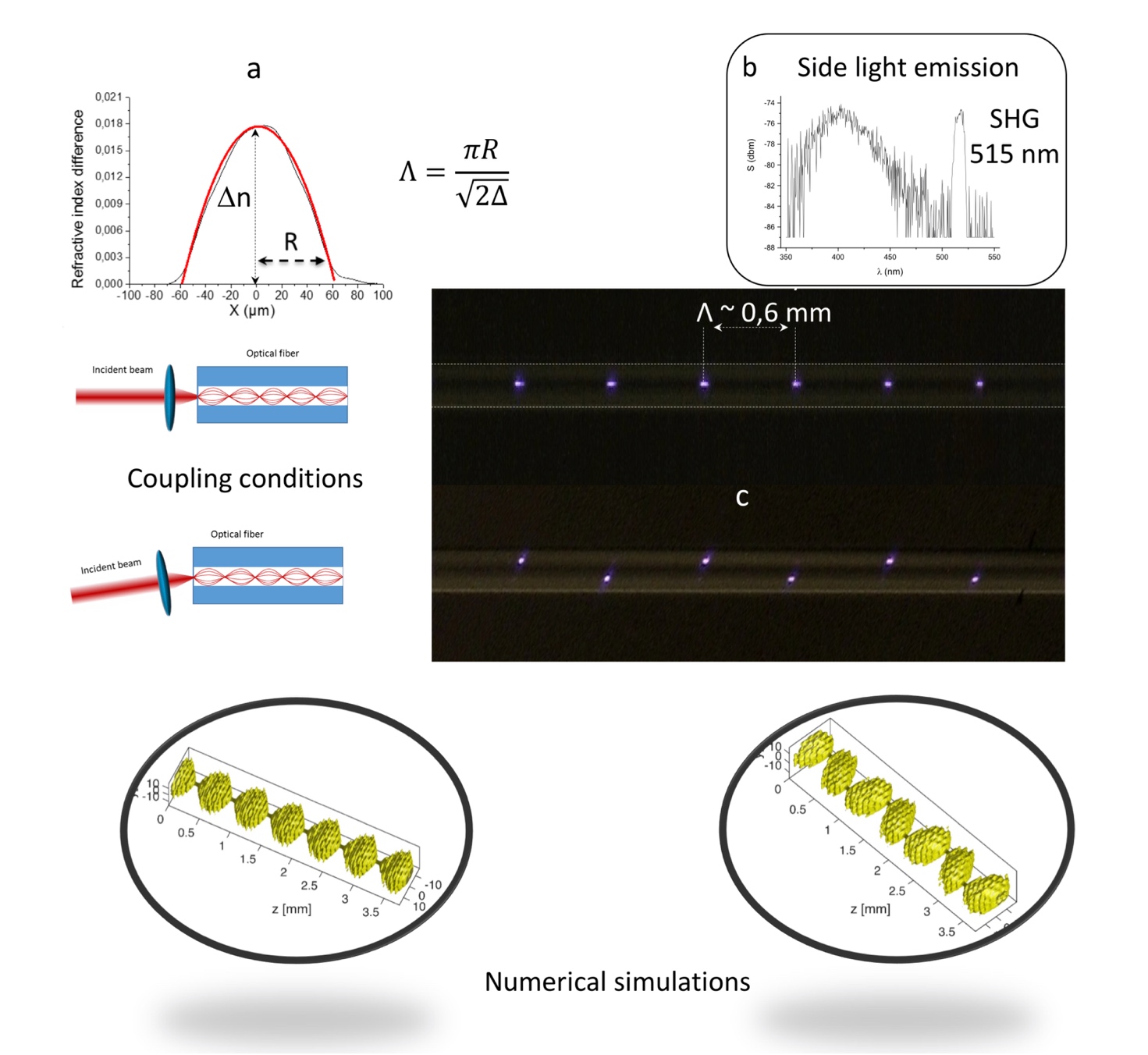
**

**Figure SM3:** Observation of the periodic self-imaging process in a 50/125 GRIN MMF by using a non-collinear frequency conversion and Cerenkov-like process, and simultaneous blue fluorescence emission; a, parabolic index profile of the used GRIN MMF; b, spectral analysis of the emitted light on the fibre side; c, experimental image of the fibre side when excited by powerful femtosecond pulses; d, numerically calculated isointensity surface, which identifies the point of minimum waist of the beam and consequently the points of maximal light intensities (on-axis excitation (left) and an off-axis coupling (right)).


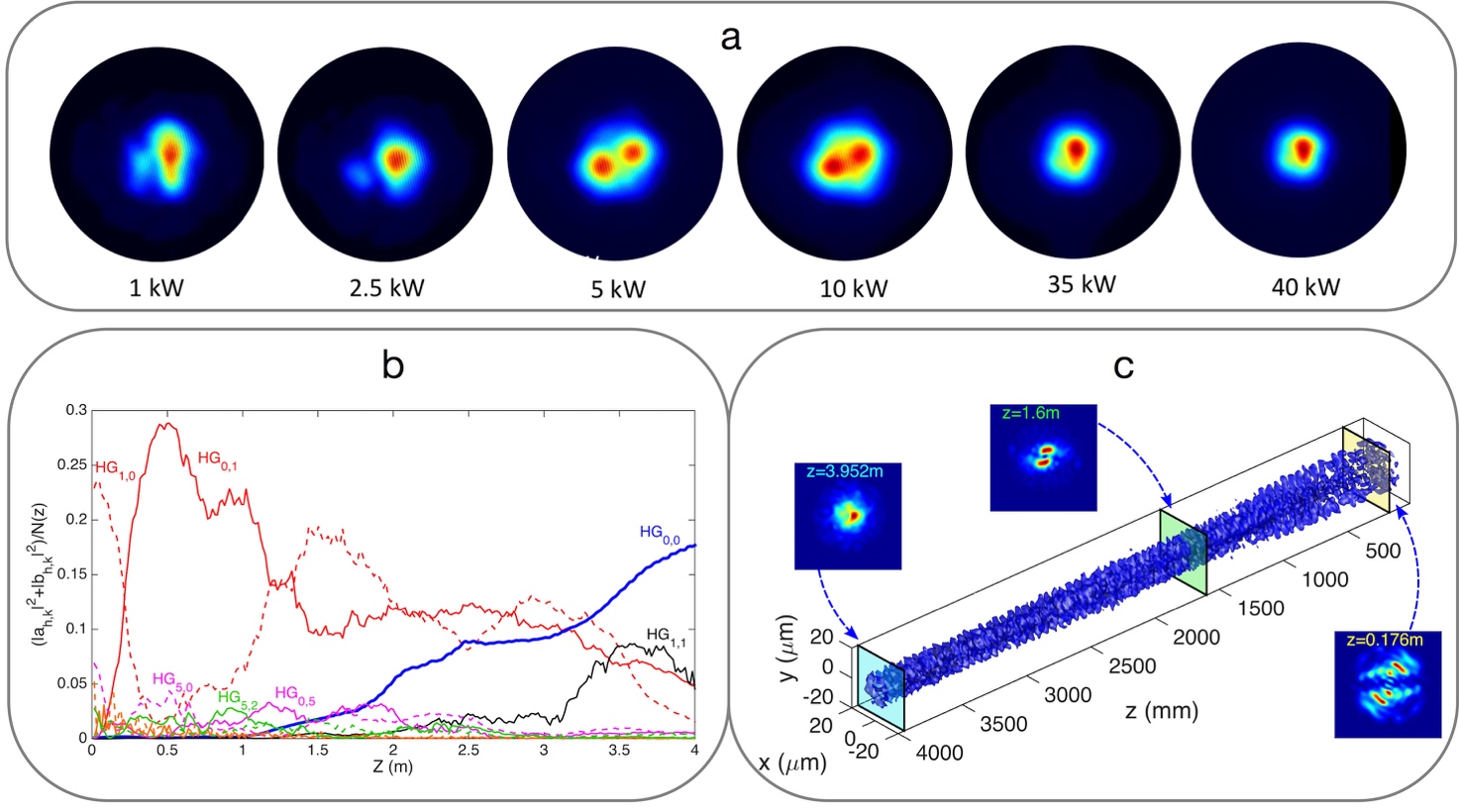


**Figure SM4:** a, Experimental analysis of output beam intensity pattern for different input peak powers in a 50/125 graded index optical fibre, with a fixed fibre length of 5 m; pump wavelength: 1064 nm, pulse duration: 740 ps. b, and c, numerical simulation for a fixed input beam condition (pulse duration: 5 ps, maximum intensity 4 GW/cm^2^, including the presence of fibre disorder, and in the absence of Raman scattering); the projections on the Hermite Gauss (HG) modes (see panel b) show that, after an initial section of dominant contribution of modes with two intensity lobes HG_0,1_, HG_1,0_ , the fractional contribution of the fundamental mode HG_0,0_ gradually increases. The corresponding isointensity surface at 50% of the maximum intensity (panel c) also shows the corresponding change in shape. These results lead to think that the process of self-cleaning towards the LP11 is not a stationary state, but rather it represents a transient state which may eventually evolve towards a beam cleaning on the fundamental LP01 mode.

**Additional comments on numerical methods**

The propagation of the complex field envelope, composed by two polarization components A and B, was numerically studied along the GRIN fibre starting from the following equations:

$$\frac{\partial A}{\partial z}=\frac{i}{2k_{0}}\nabla_{\perp}^{2}A-i\frac{\beta_{2}}{2}\frac{\partial^{2}A}{{\partial t}^{2}}-i\frac{k_{0}\Delta}{R^{2}}r^{2}A+i\gamma\left( \left| A \right|^{2}+\frac{2}{3}\left| B \right|^{2} \right)A$$

$$\frac{\partial B}{\partial z}=\frac{i}{2k_{0}}\nabla_{\perp}^{2}B-i\frac{\beta_{2}}{2}\frac{\partial^{2}B}{{\partial t}^{2}}-i\frac{k_{0}\Delta}{R^{2}}r^{2}B+i\gamma\left( \left| B \right|^{2}+\frac{2}{3}\left| A \right|^{2} \right)B (SM1)$$

The optical fibre radius is R, the relative refractive index difference between core and cladding is $\Delta,$ and the slowly varying A and B envelopes describe a field with central carrier angular frequency $\omega_{0}$ corresponding to a laser wavelength of 1064 nm; $k_{0}=\omega_{0}n_{0}/c$ and $\gamma=\omega_{0}n_{2}/c$. $\beta_{2}$ is the group velocity dispersion. These equations are valid as long as the radial coordinate r≦R; in the numerical model, the parabolic profile is truncated and fixed at the cladding value for r>R.

To mimic an effect similar to that of fibre stress or bending, we applied a coarse step beam propagation method, based on introducing a sequence of random local deformations to the refractive index profile. The coarse step is applied every 5mm. On each coarse step, the electric field is first rotated by a random angle taken from a uniform distribution. Then the fibre core is made slightly elliptical along the segment of propagation, so that the profile is described by a minimum radius r_min_ and a maximum radius r_max_ ; those values are again selected by random variables with uniform distribution and a maximum deviation of 0.1μm from the reference value R=25μm on each axis; the procedure is repeated for both polarization components. The ellipse describing the local deformation of the fibre core is also randomly oriented with an angle $\phi$. Moreover, the ellipse orientation as well as r_min_ and r_max_ assume different values for the two polarization components A, B, so that at each coarse step the waveguide contributions are calculated with slightly different refractive index profiles n_A_ and n_B_. In summary, on each coarse step the refractive index profiles are functions of the following arguments: $n_{A}\left( r_{min,A},r_{max,A},\Delta,n_{core},n_{clad,}\phi_{A} \right)$ and $n_{B}\left( r_{min,B},r_{max,B},\Delta,n_{core},n_{clad,}\phi_{B} \right)$. Optionally the maximum refractive index of one of the axis can be slightly modified to consider further effects of birefringence.

The input condition is a Gaussian beam, with a diameter FWHMI of 40 μm, pulse duration of 5 ps, peak intensity of 5 GW/cm^2^. The simulated GRIN fibre has n_core_=1.47, n_clad_=1.457. The value of the group velocity dispersion, when considered a fixed parameter, was$\beta_{2}=16.55\times{10}^{-27}s^{2}/m$, and the simulation was extended up to 4 m of propagation (figure SM4). The presence of the random variation of the fibre shape along the propagation, and the choice of a large input gaussian beam gradually leads to the formation of speckles along the propagation.

In the case of the interferometer it is important to consider also the different group delays cumulated by the multimodal waves in the two fibers, as this effect has a direct consequence in the interferogram. For the case of figure 3 of the main text we have then adopted an extended version of the propagation model that is easier to formulate for the temporal frequency spectra of the two polarization fields $\tilde{A}\left( x,y,\omega\right), \tilde{B}\left( x,y,\omega\right)$ as follows:

$$\frac{\partial\tilde{A}}{\partial z}=\frac{i}{2k\left( \omega\right)}\nabla_{\perp}^{2}\tilde{A}+iD(\omega)\tilde{A}-ik(\omega)\Delta\frac{r^{2}}{R^{2}}\tilde{A}+i\gamma\mathcal{F}\left[ \left( \left| A \right|^{2}+\frac{2}{3}\left| B \right|^{2} \right)A \right]$$

$$\frac{\partial\tilde{B}}{\partial z}=\frac{i}{2k\left( \omega\right)}\nabla_{\perp}^{2}\tilde{B}+iD\left( \omega\right)\tilde{B}-ik\left( \omega\right)\Delta\frac{r^{2}}{R^{2}}\tilde{B}+i\gamma\mathcal{F}\left[ \left( \left| B \right|^{2}+\frac{2}{3}\left| A \right|^{2} \right)B \right] (SM2)$$

Where $k(\omega)=\omega n(\omega)/c$, and $D(\omega)$ accounts for the germanium doped glass dispersion, and can be also well approximated by a constant group velocity dispersion in the spectral window considered for the simulations. The operator $\mathcal{F}\left[ \cdot\right]$ here denotes the temporal Fourier transform. The coarse step method based on the elliptical deformation of the fibre core has then been applied to eqs. SM2 to have a more reliable prediction of the speckle formation and the interference pattern. Figure 3 of the main text was obtained with a pulse duration of 10 ps, input beam diameter FWHMI of 40 μm, and peak intensity of 5 GW/cm2; the simulation was extended to 1.5 m.

The two arms of the interferometer are simulated by using two different random noise seeds, in order to obtain two different sequences of fibre deformations: the same input beam leads then two different vector complex fields at the output of the two fibers. The fringe pattern is obtained by post processing the numerical results: the coherent superposition of the two output vector fields is calculated by adding a spatial frequency shift in one of the two arms. The fringe pattern and the dispersive spectrogram are finally obtained by selecting one polarization component.

**References**

1. Efimov, A, Different measures of speckle and coherence at the output of a multimode optical fibre. *Journ. Opt. Soc. Am. A* **36**, 1-11 (2019).
2. Zerne, R. et al, Phase-Locked High-Order Harmonic Sources. *Phys. Rev. Lett.* **79**, 1006-1009 (1997).
3. Kazansky, P.G., et al., Anomalous Anisotropic Light Scattering in Ge-Doped Silica Glass. *Phys. Rev. Lett*. **82**, 2199-2202 (1999)
4. Deliancourt, E. et al. Kerr beam auto-selection of a low order mode in graded-index multimode fibre. *OSA Continuum* **2**, 1089-1096 (2019).
5. Deliancourt, E. et al. Wavefront shaping for optimized many-mode Kerr beam self-cleaning in graded-index multimode fibre. *Opt. Express* **27**, 17311-17321 (2019).
6. Zhu, X. et al. Detailed investigation of self-imaging in largecore multimode optical fibres for application in fibre lasers and amplifiers. *Opt. Express* **16**, 16632-16645 (2008).
7. Krupa, K. et al. Spatial beam self-cleaning in multimode fibres. *Nat. Photonics*, **11**, 237–241 (2017).
